# Enhancement of oral bioavailability of quercetin by metabolic inhibitory nanosuspensions compared to conventional nanosuspensions

**DOI:** 10.1080/10717544.2021.1927244

**Published:** 2021-06-18

**Authors:** Haowen Li, Manzhen Li, Jingxin Fu, Hui Ao, Weihua Wang, Xiangtao Wang

**Affiliations:** Institute of Medicinal Plant Development, Chinese Academy of Medical Sciences & Peking Union Medical College, Haidian District, Beijing, PR China

**Keywords:** Quercetin, nanosuspensions, metabolic inhibition, pharmacokinetic, oral bioavailability

## Abstract

Quercetin-loaded nanosuspensions (Que-NSps) added metabolic inhibitors were evaluated as drug delivery system to promote the oral bioavailability of quercetin. Que-NSps were prepared respectively using d-alpha tocopherol acid polyethylene glycol succinate (TPGS) or Soybean Lecithin (SPC) as stabilizer. On the basis, Piperine (Pip) or sodium oleate (SO) was, respectively, encapsulated in Que-NSps as phase II metabolic inhibitors. The resulting Que-NSps all displayed a mean particle size of about 200 nm and drug loading content was in the range of 22.3–27.8%. The release of quercetin from Que-NSps was slow and sustained. After oral administration of 50 mg/kg different Que-NSps, the levels of free quercetin in plasma were significantly promoted, the concentration of quercetin metabolites (isorhamnetin and quercetin 3-O-β-d-Glucuronide) were decreased. The absolute bioavailability was, respectively 15.55%, 6.93%, 12.38%, and 23.58% for TPGS-Que-NSps, TPGS-SO-Que-NSps, SPC-Que-NSps, and SPC-Pip-Que-NSps, and 3.61% for quercetin water suspension. SPC-Pip-Que-NSps turned out to an ideal nanocarrier combined nano drug delivery system together with metabolic inhibitor to promote oral absorption of quercetin.

## Introduction

**Figure 1.**
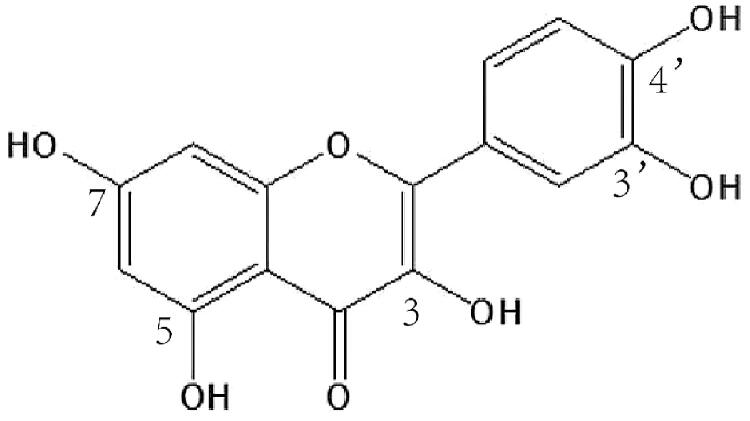
The chemical structure of quercetin.

Quercetin (Que, 3,3′,4′,5,7-pentahydroxyflavone, Figure 1) is a typical polyphenol flavonoid widely found in various fruits, vegetables and grains, accounting for 65-70% of flavanols intake in human diet (Almaghrabi, 2015). It is almost insoluble in water (about 0.09 μg/mL in water, 5.5 µg/mL artificial gastric juice and 28.9 µg/mL in artificial intestinal juice) (Xiao et al., 2005). Quercetin demonstrates an extensive range of pharmacological activities, for example, as a potent antioxidant agent, it can scavenge reactive oxide species (ROS) and superoxide anions effectively (Shahidi and Ambigaipalan, 2015). Quercetin exerts antihypertensive activity by reducing oxidative stress, improving renin–angiotensin–aldosterone system (RAAS) and vascular function (Larson et al., 2012). It can also protect myocardial mitochondrial enzyme from drug induced cardiomyocytes injury (Guzy et al., 2003). Many investigations indicated that quercetin has excellent anti-tumor effect (Rauf et al., 2018). Quercetin can inhibit the proliferation of various tumor cells, induce tumor cell apoptosis, inhibit cell cycle, and promote the release of matrix metalloproteinases (Perez et al., 2014; Lan et al., 2017). Except for inhibiting the growth of tumor cells, quercetin can also improve tumor microenvironment and up-regulate the sensitivity of tumor cells to chemotherapy drugs when administered together with chemotherapeutics (Lei et al., 2018; Ma et al., 2019). Due to its beneficial effects on human health, quercetin has attracted much medicinal interests.

However, like most flavonoids, quercetin has very low oral bioavailability which greatly limits its application as a clinically therapeutic agent (Bhattaram et al., 2002; Fasolo et al., 2007; Kumari et al., 2010). The most important reason is the water insoluble property of quercetin. On the one hand, the gastrointestinal tract is surrounded by a mucus layer with 90% water content (Macierzanka et al., 2011). Although quercetin can easily permeate through the phospholipid bilayer of Caco-2 cells in intestinal tract due to its hydrophobic nature (Nait Chabane et al., 2009), it is unable to pass though the mucus layer to reach the intestinal cells and be absorbed. On the other hand, quercetin will be rapidly metabolized once absorbed into enterocytes and blood. Researches have proved that the main metabolic pathways of quercetin were glucuronidation (the principal phase II metabolism through uridine diphosphate-glucuronic acid transferase (UGT) in gastrointestinal tract), sulfation, and methylation (Day et al., 2001; Mullen et al., 2006; Boots et al., 2008; Federica et al., 2008). Quercetin aglycone is commonly not found in plasma but its glucuronic acid, sulfate, or methyl conjugates were exclusively present in plasma (Williamson and Manach, 2005). It was reported that the absorbed quercetin in healthy volunteers was between 3% and 17% after administered 100 mg/kg of quercetin (Simioni et al., 2018). Only 20% of total quercetin was absorbed in rats given radiolabeled quercetin by gavage whatever it was free quercetin or metabolized quercetin (Xiao et al., 2005). After a single oral treatment of rats with 10 mg quercetin/200 g body weight, 93% of quercetin was metabolized an hour later (Justino et al., 2004). Some researches demonstrated that the *in vivo* physiological activity of the major metabolites like quercetin glucosides are much weaker than quercetin (Federica et al., 2008, 2009; Menendez et al., 2011), thus, enhancing the oral bioavailability of quercetin aglycone is a crucial factor for the drug efficacy of quercetin.

To sum up, effective approaches to increase quercetin bioavailability rely on increasing its hydrophilicity. A number of novel nano formulations have emerged in recent years to improve oral absorption and bioavailability of poorly soluble drugs like quercetin (Cai et al., 2013), among which cyclodextrin inclusion, liposomes, micelles, and nanosuspensions are representative ones. For example, Kale et al. (2006) prepared SBE7-β-cyclodextrin inclusion complex of quercetin, which significantly improved the dissolution rate of quercetin by 1.9 times. In another research, quercetin nano-system (QCN) prepared using Eudragit®E and polyvinyl alcohol (PVA) as carrier significantly increased the release rate of quercetin by 74 times compared to crude drug (Wu et al., 2008). Li et al. (2009) prepared quercetin solid lipid nanoparticles using soybean lecithin, Tween 80, and PEG 400, which increased AUC _(0-48 h)_ (area under the curve) of quercetin by 5.71 times in oral pharmacokinetics study (50 mg/kg, BW). These formulations effectively solved the poor solubility problem of quercetin, and increased quercetin dissolution. However, oral bioavailability of quercetin was still limited, largely due to the extensive metabolism in gastrointestinal tract unchanged.

Currently, the pharmaceutical excipients (PEs) with metabolic enzyme inhibition activities (e.g. TPGS, sodium oleate (SO) and cremophor EL) were used in nanoemulsions and effectively enhanced oral absorption of some drugs susceptible to intestinal metabolism (Zhou et al., 2015; Zhang et al., 2016). TPGS is considered as one of the most ideal pharmaceutical excipients for nano-size oral drug delivery systems. It has been reported to improve drug permeability through cell membrane and enhance cellular uptake by suppress P-glycoprotein-mediated multi-drug resistance, and thus increase the oral bioavailability of poorly soluble drugs and prolong circulation time of the coated nanoparticles (Mei et al., 2013; Zhu et al., 2014, 2016). Dong et al. tested 21 PEs for the modulation of chrysin glucuronidation, and found that five PEs significantly inhibited chrysin glucuronidation, among which sodium oleate was the most potent inhibitor. Considering the similarity of chemical structure of chrysin and quercetin, sodium oleate may be also effective in reduction quercetin glucuronidation in intestinal tract and thus increased the oral availability of quercetin (Dong et al., 2017). In addition to PEs, enzyme inhibitory compounds have also been used to increase the oral availability of drugs suffering from intestinal metabolism. Piperine (Pip) is the first globally recognized bioavailability enhancer for many drugs by inhibiting P-g protein and the cytochrome P450 (Bhardwaj et al., 2002; Volak et al., 2008). For example, Bi’s study demonstrated that piperine as a bioenhancer increased the bioavailability of silybin to 146–181% (Bi et al., 2019). In another study, the area under the curve (AUC_0→∞_) of 20(S)-protopanaxadiol (PPD)-cubic nanoparticles containing piperine was 2.48 times that of PPD and 1.46 times that of PPD-Cubic nanoparticles (Jin et al., 2013).

However, nearly all the related researches employed nanoemulsions as drug delivery system. Nanoemulsions usually need excessive amount of surfactants and other excipients with very low drug-loading content, the former can cause potential damage to the intestinal mucosa especially after long time use, while the latter is unsuitable for natural products such as flavonoids that require large dosage.

In this study, quercetin was fabricated into nanosuspensions, which have great advantages in drug-loading content and suitable for the requirement of large dose for *in vivo* drug delivery, and sodium oleate or piperine was incorporated to verify if metabolic inhibitory PEs or partner molecules could effectively enhance the oral availability of quercetin nanosuspensions.

## Materials and methods

### Materials

Quercetin was purchased from Beijing Ouhe Technology Co., Ltd. (Beijing, China). SPC was purchased from Shenyang Tianfeng Pharmaceutical Co., Ltd. (Shenyang, China). TPGS was purchased from Xi’an Healthful Biotechnology Co. Ltd. (Xi’an, China). Sodium oleate were bought from BioRuler Co. Ltd. (Rockville, MD) Piperine was purchased from Nanjing DASF biotechnology Co. Ltd. (Nanjing, China). zirconium beads (TZP beads, 0.4-0.6 mm) were bought from Beijing Xinmei Hongxin Technology Co., Ltd. (Beijing, China). Acetonitrile was high-performance liquid chromatography (HPLC) grade purchased from Fisher Scientific (Pittsburgh, PA). All other reagents were of analytical grade. Deionized water was used in all the experiments.

### Animals

Male Sprague-Dawley (SD) rats (200 ± 20 g) were bought from Vital River Lab-oratory Animal Technology Co., Ltd. (Beijing, China). All mice were provided with a 60% humidity under 12 h light–dark cycle conditions at and 25 °C for 7 days before experiments. The animal experiments followed the guidelines for Ethical and Regulatory for Animal Experiments of The Institute of Medicinal Plant Development (IMPLAD, license no. SLXD-20190702001), China.

### Preparation of Que-NSps

Que-NSps were prepared using two different methods, antisolvent precipitation method and TZP grinding method. For antisolvent precipitation method, 10 mg of Que crystalline powder was dissolved in 0.5 mL of ethanol to get an organic solution, 10 mg of stabilizer (TPGS or SPC) was dissolved in 10 mL of water. Then the organic phase was slowly injected into the water phase at 25 °C under 250 W ultrasonication. Next, the organic solvent was removed by evaporation under vacuum at 40 °C followed by homogenization at 25 °C for 10 cycles under 1500 bar to obtain two common Que-NSps (TPGS-Que-NSps and SPC-Que-NSps). For Pip-Que-NSps, 5 mg of Pip was co-dissolved with Que, 5 mg of stabilizer (TPGS or SPC) was dissolved in water. For SO-Que-NSps, 5 mg of SO and 5 mg of stabilizer (TPGS or SPC) were co-dissolved in water, the following steps were the same as above described.

For TZP beads grinding method, 10 mg of Que and 10 mg of TPGS (or SPC) were co-dispersed in 5 mL of water in a vial with a magnetic stir bar. Then appropriate amount of TZP beads was added until the height of TZP beads was same to the liquid level. The mixture was stirred at 300 rpm in ice bath for 6 h. Finally, TZP beads were removed to obtain two common Que-NSps (TPGS-Que-NSps and SPC-Que-NSps).

For the preparation of SO-Que-NSps, 10 mg of Que, 5 mg of TPGS (or SPC) and 5 mg of SO were co-dispersed in 5 mL of water in a vial with a magnetic stir bar, TZP beads were added for grinding according the same procedure as above mentioned, then, TPGS-SO-Que-NSps and SPC-SO-Que-NSps were obtained.

For the preparation of Pip-Que-NSps, 10 mg of Que, 5 mg of TPGS (or SPC) and 5 mg of Pip were co-dispersed in 5 mL of water in a vial with a magnetic stir bar, TZP beads were added for grinding according the same procedure as above mentioned, then, TPGS-Pip-Que-NSps and SPC-Pip-Que-NSps were obtained.

### Physicochemical characterizations of Que-NSps

#### Particle size measurement

The mean particle size, polydispersity index (PDI), and zeta potential of Que-NSps were measured using a dynamic light scattering (DLS, Zetasizer Nano ZS, Malvern Instruments, Malvern, UK) under room temperature.

#### Morphological observation

The morphology of Que-NSps was observed using a JEM-1400 transmission electron microscope (JEOL, Tokyo, Japan). About 5 µl of each sample was dropped on a 300-mesh copper grid, air-dried, then colored with 2% (w/v) uranyl acetate for observation under the microscope.

#### X-ray diffraction (XRD) detection

XRD patterns of different samples (Que-NSps lyophilized powder, Que bulk powder, TPGS, SPC, SO, Pip, physical mixture 1 (Que bulk powder, Pip and SPC) and physical mixture 2 (Que bulk powder, SO and TPGS) were detected using an X-ray diffractometer (DX-2700, China). Cu-Ka radiation generator set at 100 mA and 45 kV. All samples were scanned over an angular range of 3–80°, with a step size of 0.02 and a count time of 3 s per step.

#### Differential scanning calorimetry (DSC) detection

DSC thermal profiles of all powder samples (the same as samples in XRD detection) were detected by a differential scanning calorimeter (Q200, TA Instruments, New Castle, DE). About 5 mg of each sample put and sealed in standard aluminum pans was measured from 0 °C to 350 °C with a scanning rate of 10 °C/min under nitrogen environment.

#### High-performance liquid chromatography (HPLC) analysis

The concentration of quercetin was determined by an HPLC apparatus (DIONEX Ultimate 3000, Germering, Germany). A Symmetry C18 column (4.6 mm × 250 mm, 5 mm; Agilent Technologies, San Deigo, IL) was used for chromatographic separation at 30 °C. The mobile phase was composed of acetonitrile and 0.1% formic acid (40:60, v/v). The flow rate was 0.8 mL/min. The UV detection wavelength was 366 nm (UV detector, DIONEX, Sunnyvale, CA).

The methods provided good linearity (*R*^2^ = 0.9996) over a concentration range of 2–200 µg/mL with the intra-day and inter-day precision of less than 2.0% and 1.0%, respectively, and LOQ of 200 ng/mL.

#### Drug loading content measurement (DLC)

Specific weight of lyophilized Que-NSps was dissolved in a certain amount of methanol to determine DLC. The concentration of quercetin was determined by HPLC. The DLC was calculated by followed equation:

(1) DLC(%)=V×C/W×100%

where *V* is the methanol volume, *C* is the quercetin concentration, and *W* is the weight of Que-NSps.

#### Stability of Que-NSps in artificial gastric and intestinal juice

The *in vitro* stability investigation of Que-NSps in gastric or intestinal juice was performed to examine the suitability of Que-NSps for oral administration. Que-NSps were respectively well mixed with artificial gastric or intestinal fluid (1:4, v/v), followed by incubation at 37 °C. About 1 mL of the incubated mixture was taken out and measured for particle size change at specific time intervals. And the concentration changes of Que in gastric and intestinal juice were determined using HPLC at specific time intervals. Each experiment was conducted in triplicate.

#### *In vitro* drug release behavior of Que-NSps

*In vitro* behavior of drug release from Que-NSps was performed as follows. PBS (pH 7.4), artificial gastric juice (pH 1.2) and artificial intestinal juice (pH 6.8) containing 0.1% (w/v) Tween 80 (pH 7.4) were chosen as release medium respectively. Que-NSps (2 mL, 5 mg/mL of equivalent Que for PBS media) were sealed in dialysis tubes (molecular weight cut off (MWCO: 8000–10,000, Sigma, Chicago, USA). The dialysis tubes were immersed into 1 L of dissolution medium and incubated at 37 °C under continuous stirring (150 rpm).When artificial gastrointestinal juice were as release media, the lyophilized Que-NSps were dispersed in gastric juice directly (final concentration of Que: 2 mg/mL, 2 mL), immersed into 1 L of artificial gastric juice at the first 4 h and then transferred to 1 L of intestinal juice until 24 h to simulate the gastrointestinal environment and gastric emptying time *in vivo.* About 50 µL of internal liquid was taken out from the dialysis tubes at special time intervals, and the same volume of fresh release medium were replenished into the dialysis tubes. The dissolution medium was renewed every 24 h. The concentration of quercetin was analyzed by HPLC. The above experiments were performed in triplicate.

#### *In vivo* pharmacokinetic study in SD rats

For the pharmacokinetic study, the jugular vein of the rats was cannulated first through a small surgery. Then the rats were housed individually under normal conditions for 24 h, fasted for 18 h with free access to and randomly divided into six groups: (1) physical suspensions of quercetin (Que-susp, *i.g.,* quercetin was suspended in pure water containing 0.4% CMC-Na and sonicated for 30 min, well shaken before dose), (2) quercetin solution (quercetin was dissolved in a mixture of PEG400 and water (60:40 v/v; Que-sol, *i.v.*), (3) SPC-Que-NSps (*i.g.*), (4) SPC-Pip-Que-NSps (*i.g.*), (5) TPGS-Que-NSps (*i.g.*). (6) TPGS-SO-Que-NSps (*i.g.*). The dose was 50 mg/kg body weight for all groups (no matter orally or intravenously).

About 400 µL of blood was withdrawn from the jugular vein cannula at specific time intervals and then placed into heparinized tubes and separated immediately by centrifugation (5000 rpm for 10 min). The plasma obtained was stored at −80 °C until analysis. About 100 µL of plasma was respectively added 10 µL of internal standard and 690 µl of extract solution (ethyl acetate: acetone 10:1, v/v), vortexed for 30 s, then centrifuged for 10 min (13000 rpm). About 650 µL of the supernatant was transferred to a 2 mL Eppendorf tube. The sediment was added 690 µL of extract solution, vortexed, and centrifuged again. Another 650 µL of the supernatant was transferred to the same tube. Then the solvent of the supernatant was removed using a vacuum concentrator. The collected samples were dissolved in 100 µL of 50% methanol–water before concentration analysis.

### Ultra-high performance liquid chromatography-mass spectrometry (UPLC-MS-MS)

Quercetin concentration in plasma of *in vivo* pharmacokinetic studies was analyzed by UPLC-MS method (Waters Acquity I CLASS/ABSCIEX QTRAT-4500, Milford, MA). Chromatographic separation was carried out on a ACQUITY UPLC HSS T3 column (50 mm × 2.1 mm, 1.8 µm) with a ACQUITY UPLC HSS T3 guard pre-column (5.0 mm × 2.1 mm, 1.8 µL, 100 A, 3/pkg, Waters, Milford, MA). The mobile phase consisted of a gradient mobile phase system consisting of methanol containing 0.1% formic acid (phase A) and water containing 0.1% formic acid (phase B) at a flow rate of 0.2 mL /min. The pump was programed as follows: 0-0.5 min, 70% phase B; 0.5-2.5 min, 70-35% phase B; 2.5-4.5 min, 35-15% phase B; 4.5-6 min, 15-0% phase B; 6-7 min, 0% phase B; 7-7.1 min, 70% phase B; 7.1-10 min, 70% phase B. A 10 µl sample was injected into the system and column temperature maintained at 30 °C.

The mass spectrometer worked in the negative ion mode. The MS parameters were as follows: the ion spray voltage was set at −4.5 kV, and the source temperature was set at 500 °C. The curtain gas was 30 psi. The ion source gas1 and ion source gas 2 were both set at 50 psi respectively. The multiple reaction monitoring transitions were performed at *m*/*z* 3 0 1→151 for quercetin, *m*/*z* 3 1 5→300.1 for isorhamnetin, *m*/*z* 477 → 301 for quercetin 3-O-β-D-Glucuronide (Que-glu) and *m*/*z* 4 1 7→122 for nimodipine (internal standard).

### Statistical analysis

Pharmacokinetic parameters were evaluated using Phoenix WinNonlin program version 97 (SPSS Inc., Chicago, IL). Statistical analysis of experimental data was calculated by independent-samples *T*-test and *F*-test using IBM SPSS Statistics software, Version 21 (IBM Corporation, Cary, NC). *p* < 0.05 was considered as the minimal level of significance.

## Results and discussions

### Formulation optimization of Que-NSps

Stabilizer and preparation method are two important factors that influence particle size and stability of nanosuspensions. TPGS and SPC were tried as stabilizers. Then different metabolic inhibitions were added, respectively, to the formulation. Drug/stabilizer (and inhibitor) ratio was set as 1:1 (w/w). Different combination of stabilizers and metabolic inhibitors was screened. Antisolvent precipitation method followed by 15 cycles homogenization and TZP beads grinding method were tried. All Que-NSps prepared by TZP beads grinding method had smaller particle size compared to antisolvent precipitation method (Table 1). The size distribution (PDI value) was better too. TZP beads grinding method was simpler, more convenient without homogenization compared to antisolvent precipitation method. So TZP beads grinding method was chosen to prepare Que-NSps in the following research.

**Table 1:** The particle size and PDI value of Que-NSps prepared by antisolvent precipitation method and TZP beads grinding method.

	Methods			
	antisolvent precipitation		TZP beads grinding	
Formulation	Size (nm)	PDI	Size (nm)	PDI
TPGS, Que	327.2 ± 8.3	0.34 ± 0.05	182.1 ± 1.5	0.21 ± 0.02
TPGS, SO, Que	277.5 ± 3.7	0.21 ± 0.02	165.3 ± 1.3	0.20 ± 0.04
TPGS, Pip, Que	196.0 ± 7.9	0.37 ± 0.08	190.2 ± 6.3	0.33 ± 0.04
SPC, Que	302.4 ± 4.3	0.27 ± 0.03	173.6 ± 1.6	0.19 ± 0.03
SPC, SO, Que	300.5 ± 4.5	0.35 ± 0.05	233.6 ± 5.2	0.28 ± 0.04
SPC, Pip, Que	295.8 ± 3.4	0.33 ± 0.06	207.8 ± 4.2	0.29 ± 0.02

The results are presented as the mean ± SD, *n* = 3.

TPGS-Pip-Que-NSps and SPC-SO-Que-NSps were unstable during the storage at room temperature, precipitation was observed only 4 h after preparation. Therefore, the other 4 formulations were studied in the subsequent research.

### Physicochemical characterization of Que-NSps

The mean particle size of TPGS-Que-NSps and TPGS-SO-Que-NSps (1 mg/mL of Que) obtained by TZP beads grinding method was 182.1 ± 1.5 nm and 165.3 ± 1.3 nm (Table 1) respectively. SO is a surfactant and can act as stabilizer together with TPGS. Thus, the amount of stabilizer in TPGS-SO-Que-NSps was more than that of TPGS-Que-NSps, so the size of TPGS-SO-Que-NSps was smaller.

The mean particle size of SPC-Que-NSps and SPC-Pip-Que-NSps was, respectively, 173.6 ± 1.6 nm and 207.8 ± 4.2 nm (Table 1). The additional incorporation of Pip in quercetin nanosuspensions meant more insoluble material to be encapsulated and bigger hydrophobic inner core, and this led to a little bigger particle size of SPC-Pip-Que-NSps than that of SPC-Que-NSps. The small particle size was helpful for adsorption *in vivo* (Waterman and Sutton, 2003; Sigfridsson et al., 2009).

The DLC was, respectively, 27.8% and 25.6% for TPGS-Que-NSps and TPGS-SO-Que-NSps, 22.3% and 22.6% for SPC-Que-NSps and SPC-Pip-Que-NSps. Compared to theoretical DLC of 50%, TZP beads grinding method resulted in much lower DLC due to the drug loss by absorption into the tiny holes of zirconium beads, which was inevitable during the grinding process.

**Figure 2.**
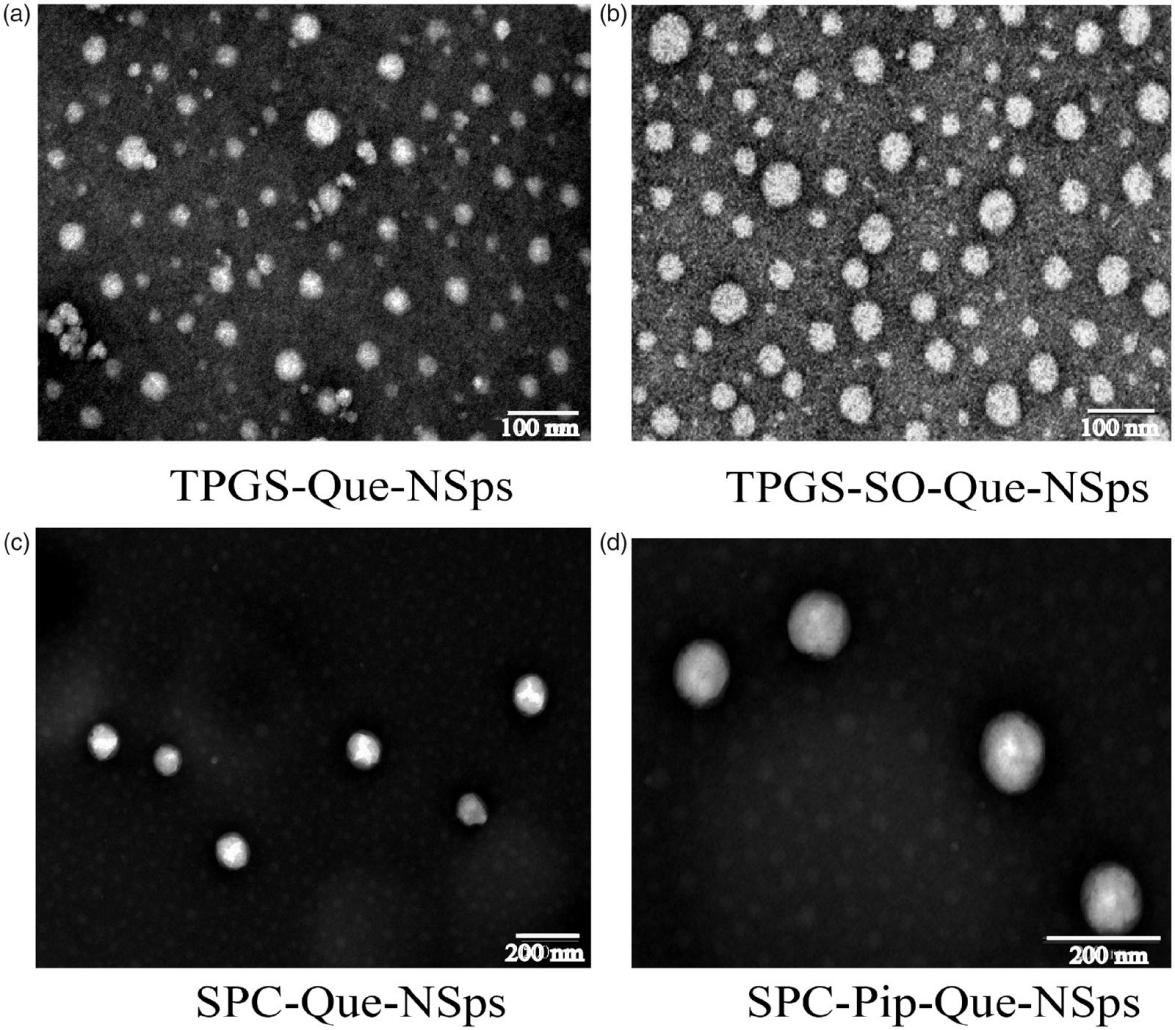
TEM image of TPGS-Que-NSps (a), TPGS-SO-Que-NSps (b), SPC-Que-NSps (c), and SPC-Pip-Que-NSps (d).

The TEM images of the 4 kind of Que-NSps are shown in Figure 2. They were all nanometer-size in spherical shape. No irregular drug crystal was visible (crystallization with the majority of stick or needle crystal in the size vary from 10 µm to a few dozen microns) (Li et al., 2009).

**Figure 3.**
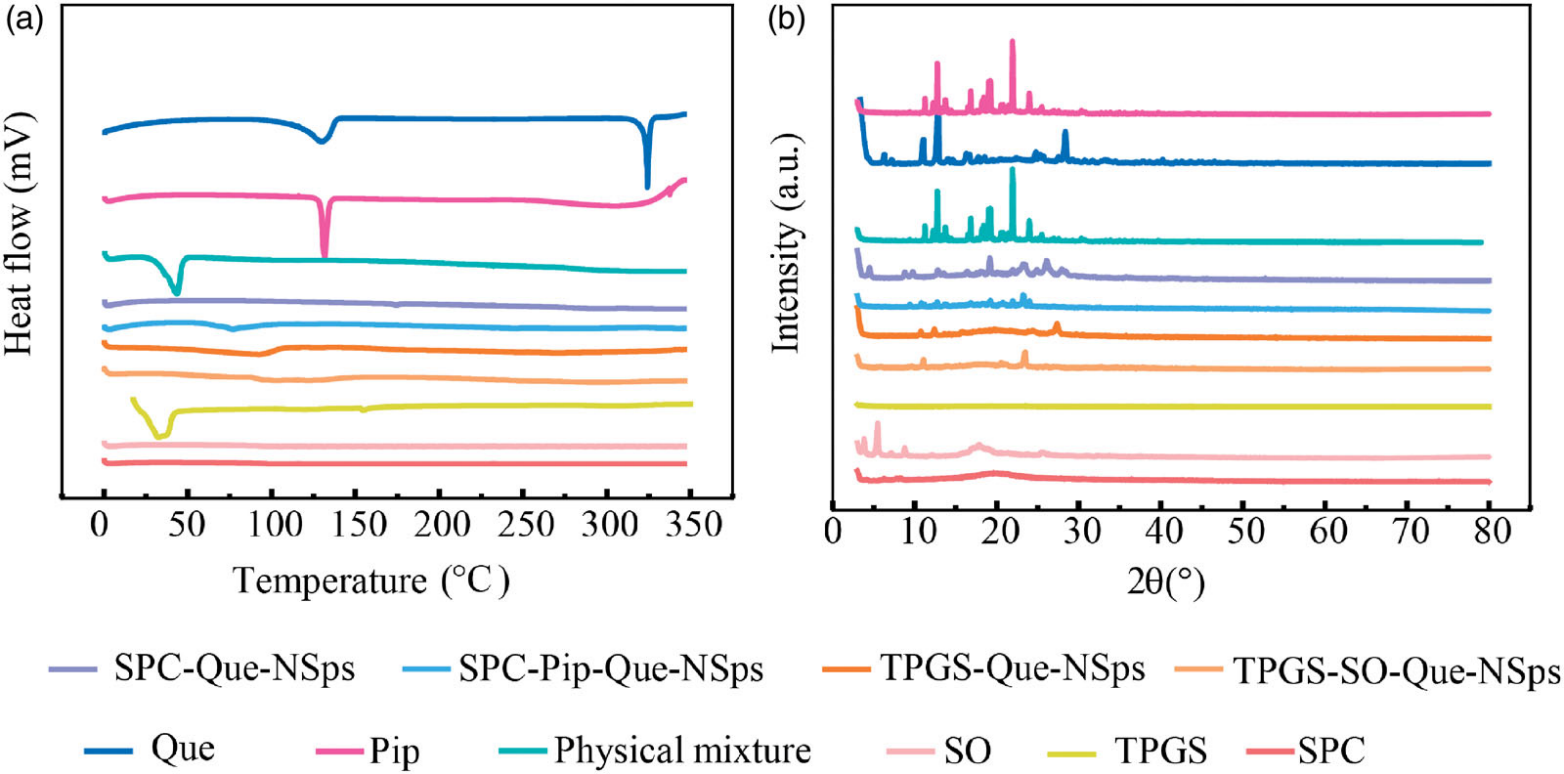
DSC thermograms and XRD patterns. (a) DSC thermograms of quercetin bulk powder, Pip, TPGS, SPC, SO, TPGS-Que-NSps, TPGS-SO-Que-NSps, SPC-Que-NSps, SPC-Pip-Que-NSps, and physical mixture (b) XRD patterns of quercetin bulk powder Pip, TPGS, SPC, SO, TPGS-Que-NSps, TPGS-SO-Que-NSps, SPC-Que-NSps, SPC-Pip-Que-NSps, and physical mixture.

The DSC investigation results are shown in Figure 3(a). Quercetin bulk powder had a water loss peak at 130 °C and a sharp melting peak at 324 °C. Pip showed an acute endothermic peak at around 130 °C which was consistent with its melting point. SPC, SO, and TPGS are polymer compounds, their melt points were low and uncertain, so they had no obvious melting peak in DSC thermogram. TPGS had a small endothermic peak at 40 °C. Their existence in the physical mixture may influence the melting peak of Que or Pip, so that the physical mixture showed no melting peak of Que. No obvious peak of Que was observed in all the nanosuspensions either.

According to XRD patterns (Figure 3(b)), Que had obvious diffraction peaks at 10.6°, 12.5°, 15.8°, 16.3°, 23.9°, and 27.4°. The XRD patterns of TPGS-Que-NSps, TPGS-SO-Que-NSps, SPC-Que-NSps, and physical mixture had consistent diffraction peaks with Que powder (signal intensity of physical mixture was weaker) indicating that queecetin existed as crystalline structure in Que-NSps. The XRD patterns of SPC-Pip-Que-NSps exhibited consistent peaks with Que and Pip, which identified the existence of Que and Pip crystalline in SPC-Pip-Que-NSps.

### Stability of Que-NSps

**Figure 4.**
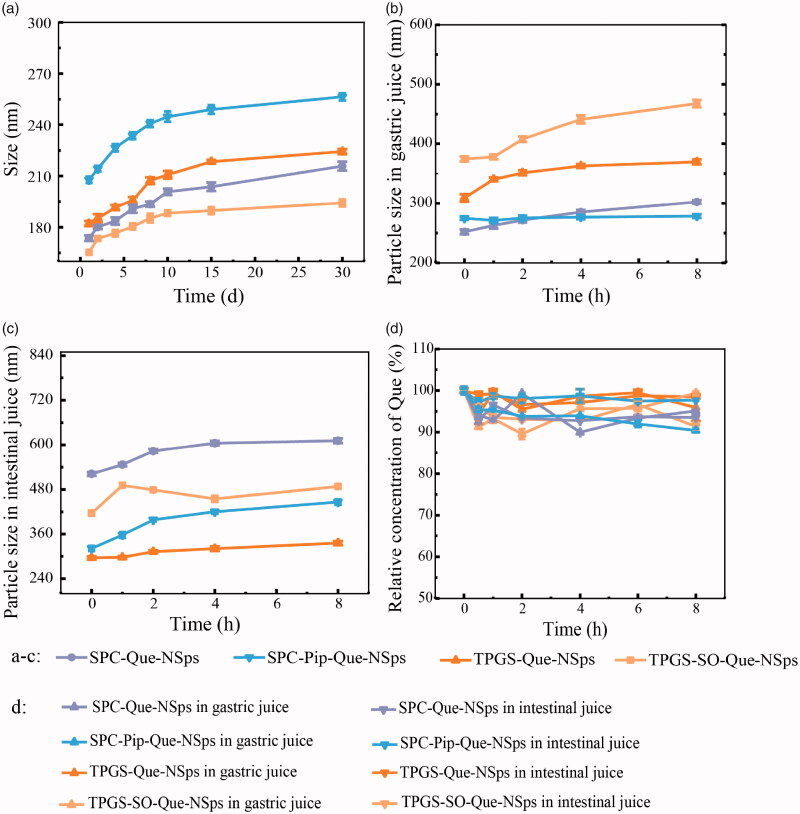
The stability of Que-NSps (mean ± SD, *n* = 3). Mean particle size change curves of Que-NSps during the storage at room temperature (a); the mean particle size change curves of Que-NSps during the incubation in artificial gastric fluid (b) or in artificial intestinal fluid (c) at 37 °C until 8 h; The concentration changes of Que in Que-NSps during the incubation in artificial gastric fluid or in artificial intestinal fluid at 37 °C until 8 h (d).

All Que-NSps were stable for more than a month without significant size increasement (Figure 4(a)). No obvious aggregation occurred.

The stability of Que-NSps incubated with artificial gastric and intestinal fluids were studied for 8 h (Figure 4(b,c)). The particle size of TPGS-Que-NSps was increased to 369.5 nm in artificial gastric fluid and 335.8 nm in artificial intestinal fluid after 8 h. The particle size of TPGS-SO-Que-NSps was bigger than TPGS-Que-NSps in gastric and intestinal fluid, respectively, 488.2 nm and 467.8 nm. The particle size of SPC-Que-NSps was increased to 302.4 nm and 611.2 nm in gastric and intestinal fluid until 8 h. SPC-Pip-Que-NSps was more stable, the particle size was separately 278.6 nm and 446 nm in gastric and intestinal fluid until 8 h. Although the particle size of 4 Que-NSps were enlarged in gastric and intestinal fluid, they were all at the nanometer level for at least 8 h.

The Que concentration of different Que-NSps incubated with artificial gastric and intestinal were also quite stable until 8 h (shown in Figure 4(d)). The concentration just fluctuated in a narrow scale and did not show obvious changes compared to the beginning Que concentration. The above indicated that all the prepared Que-NSps were suitable for oral administration.

### *In vitro* drug release behavior of Que-NSps

**Figure 5.**
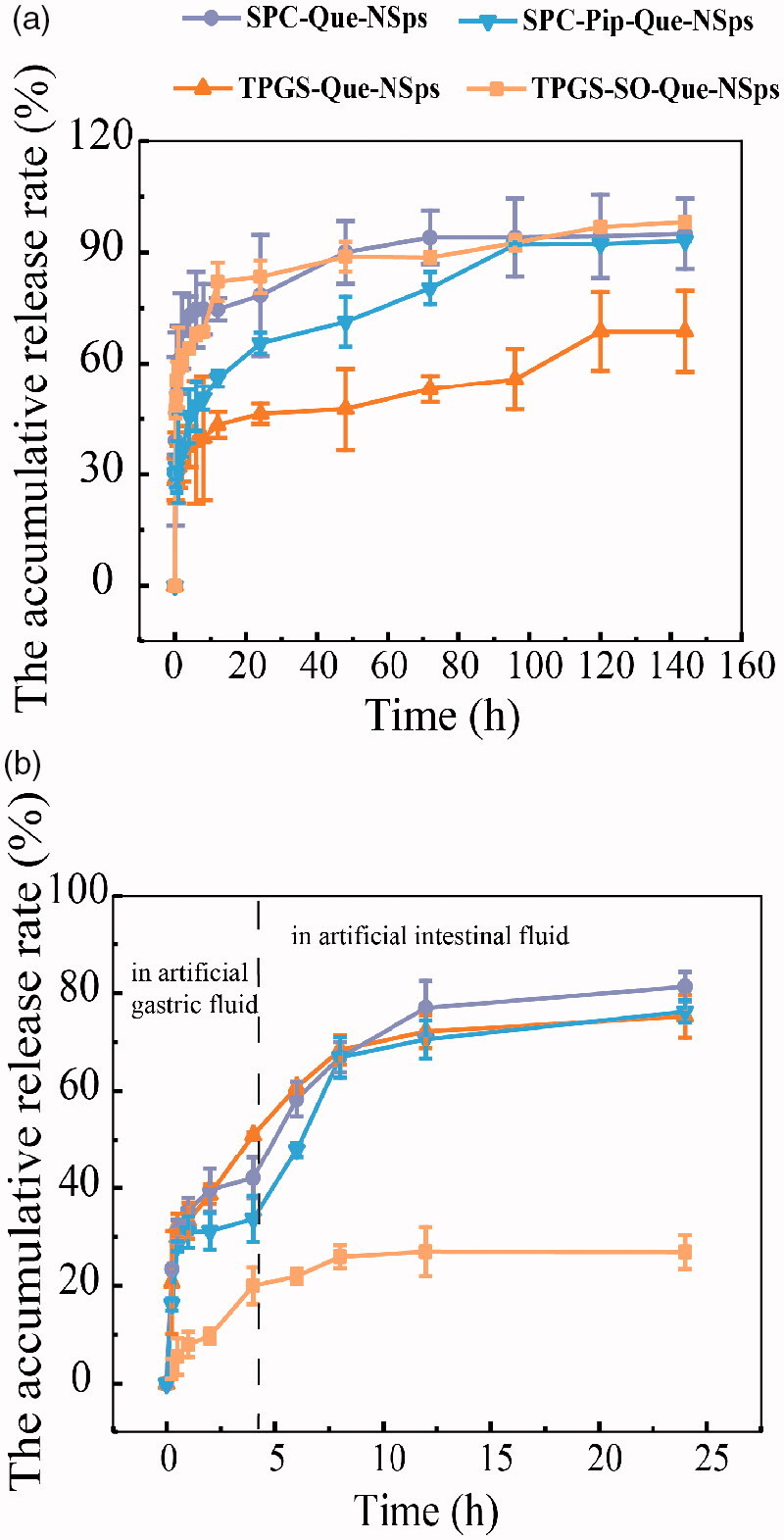
The *in vitro* drug release profiles of Que-NSps in PBS containing 0.1% (w/v) Tween 80 at 37 °C (a) and in artificial gastrointestinal juice containing 0.1% (w/v) Tween 80 at 37 °C (b) (mean ± SD, *n* = 3).

The cumulative drug release profiles of different Que-NSps are shown in Figure 5. At first, PBS containing 0.1% (w/v) Tween 80 (pH 7.4) was used as release medium. The solubility of Que in this medium reached 30 µg/mL, meeting the sink requirement for *in vitro* drug release (Figure 5(a)). SPC-Que-NSps displayed a quite quick drug release withing the first 2 h with a cumulative release up to 68.87%, then a slow release reaching a plateau of 94.01% at the 72th hour. As for SPC-Pip-Que-NSps, a burst release was also observed within 30 min, then a slower drug release reaching a plateau of 93.09% at the 96th hour. TPGS-Que-NSps also showed a burst release first, up to 28.69% within 0.17 h, then a steady and slow release, and the cumulative release reached 68.73% at 144 h.

In general, SPC-Que-NSps and TPGS-SO-Que-NSps showed very similar *in vitro* drug release pattern. Additional incorporation of insoluble Pip in the nanosuspensions slowed down the drug release, resulted in a slower release of Que within the first 96 h for SPC-Pip-Que-NSps in contrast to SPC-Que-NSps. TPGS-Que-NSps, possibly due to the π-π stacking effect between Que and the aromatic ring of the hydrophobic part of TPGS, the drug release was much slower than SPC-Que-NSps, in which no π-π stacking existed.

In most cases, *in vitro* drug release investigation was performed through the determination of the drug concentration in the dialysate. However, in our study, the drug release from quercetin nanosuspensions was very slow during some stages especially after the first renewal of release medium at 24th hour so that the determination of Que concentration in the release medium or dialysate was difficult. Meanwhile the release Que or free Que in in the release medium was not stable and easily be oxidated during 24 h of incubation at 37 °C before the next renewal of the release medium. Thus, determination of the remnant Que in the dialysis tubing, through which the above problem could be overcome, was adopted in this study.

Then, the *in vitro* drug release profiles of Que-NSp in artificial gastric juice (pH 1.2) and artificial intestinal juice (pH 6.8) were studied to simulate the gastrointestinal environment and gastric emptying time *in vivo* (Figure 5(b)). The release behaviors of Que-NSps in artificial gastrointestinal fluids were different from which in PBS media. SPC-Que-NSps and SPC-Pip-Que-NSps displayed similar drug release profiles in artificial gastrointestinal fluids. At the first 0.5 h, they both showed a burst release in gastric fluid (31.15% for SPC-Que-NSps and 28.14% for SPC-Pip-Que-NSps). Then the release rate slowed down until 4 h and the cumulative release rates were respectively 42.08% and 33.68% for SPC-Que-NSps and SPC-Pip-Que-NSps. The release rate became faster when SPC-Que-NSps and SPC-Pip-Que-NSps were transferred to intestinal fluid, the cumulative release rates were, respectively, 76.98% and 70.53% until 12 h. In the next 12 h, a slower drug release reached a plateau of 81.31% for SPC-Que-NSps and 76.24% for SPC-Pip-Que-NSps at 24 h.

For TPGS-Que-NSps, three different release phases were observed. At the first 0.5 h, there was a burst release with a cumulative release up to 32.30%. Then the release rate was slower until 8 h with a cumulative release of 68.36%, followed by an even slower drug release reaching a plateau of 75.22% until 24 h.

Quite differently from the above three quercetin nanosuspensions, TPGS-SO-Que-NSps release drug at a much lower level in artificial gastrointestinal fluids. The cumulative drug release rate was only 25.92% at 8 hour and 26.97% at the 24th hour. The main reason was that TPGS-SO-Que-NSps was not very stable in artificial gastric fluid. Aggregates were observed when TPGS-SO-Que-NSps released in gastric fluid. The aggregates adhered to the dialysis tubing and blocked the holes of the dialysis tubing, through which the released quercetin passed into the medium. The physical mixture of TPGS-Que-NSps and SO solution showed similar behavior in gastric fluid but not in intestinal fluid. So, it was the co-existence of SO and the acid pH that resulted in aggregation and adherence of TPGS-Que-NSps to the dialysis tubing. TPGS-SO-Que-NSps aggregation meantime retarded the drug release of quercetin from the nanosuspensions.

### *In vivo* pharmacokinetic studies in SD rats

**Figure 6.**
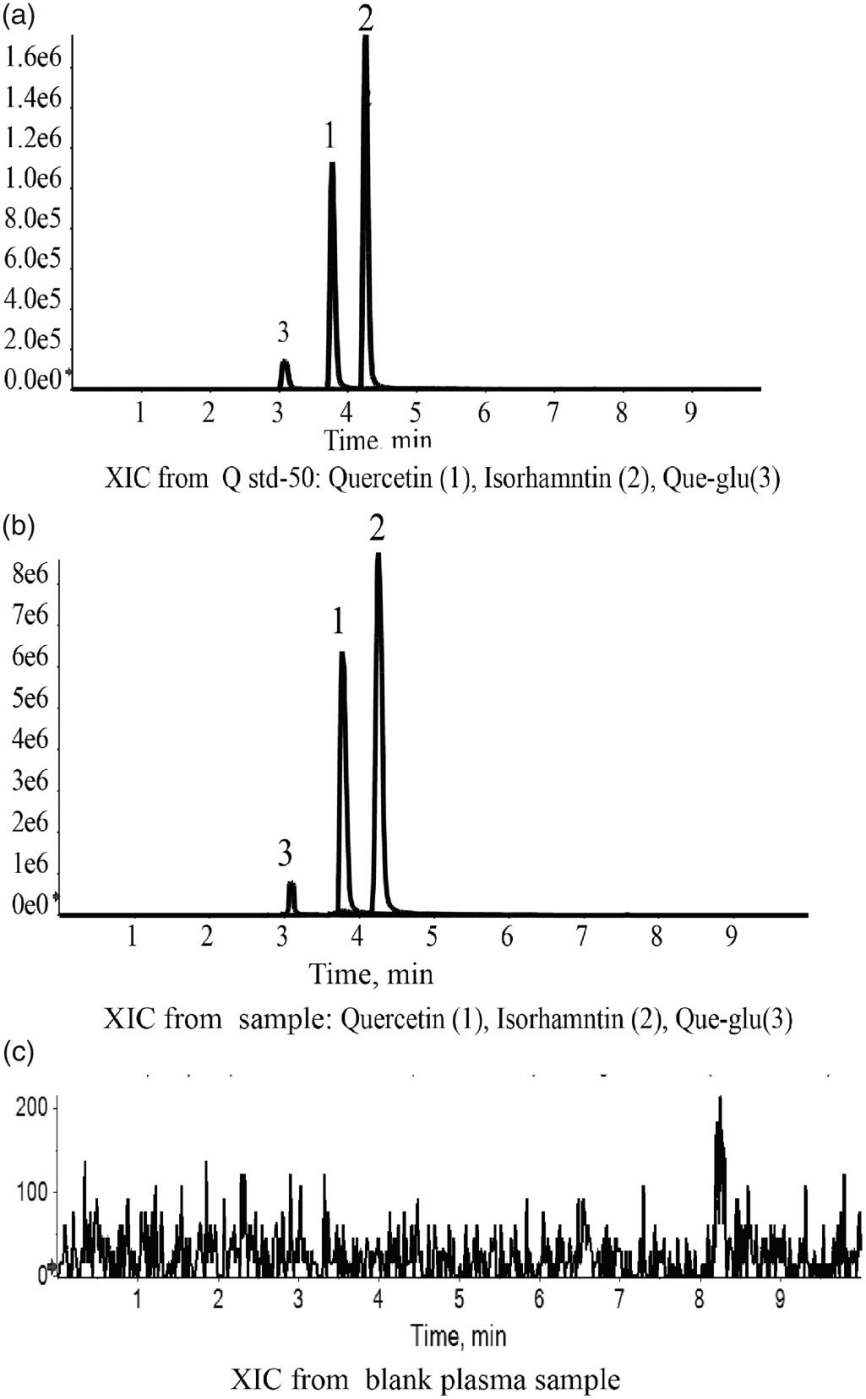
LC-MS chromatograms of Que, Iso and Que-glu in standard plasma sample (a), tested plasma sample (b), and blank plasma sample (c).

A lot of previous studies on pharmacokinetics of quercetin detected the total quercetin in plasma, including free quercetin and some metabolic products of quercetin that was transformed into quercetin through acid hydrolysis. In our research, the prototype of quercetin in plasma was directly detected. To evaluate whether different formation of nanosuspensions can inhibit gastrointestinal metabolism of quercetin or not, the main metabolites of quercetin: Que-glu and isorhamnetin were also detected (Yao et al., 2013; Liao and Lin, 2014). A modified extraction method was used to enable determination of the free quercetin, Que-glu and isorhamnetin in plasm at the same time. The chromatograms showed a stable baseline and good resolution among three target chemicals, internal standard (nimodipine), and endogenous materials in plasma (Figure 6). The limit of detection was 1 ng/mL for quercetin and isorhamnetin (1–1000 ng/mL), 5 ng/mL for Que-glu (5–1000 ng/mL) with inter-precision less than 2.0% for all of them. The method accuracy was between 85% and 115%. The relative standard deviation (RSD) at low, medium, and high concentration were all less than 15% and RSD was less than 20% at lower limit of quantitation. The mean recovery of quercetin, Que-glu and isorhamnetin in plasma was all above 90%.

**Figure 7.**
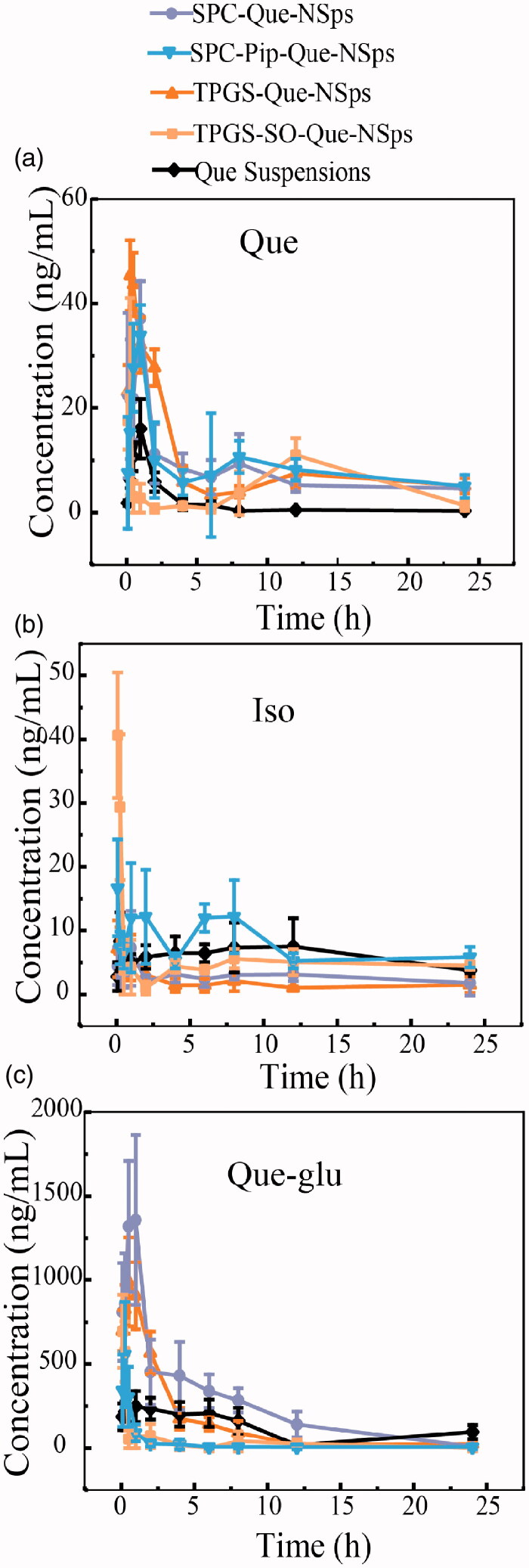
The mean plasma concentration-time curve of quercetin (a), Que-glu (b), and isorhamnetin (c) in rats after a single oral dose (50 mg/kg) of Que-NSps and Que-suspension (mean ± SD, *n* = 6).

Oral pharmacokinetic parameters of each quercetin nanosuspensions were calculated using WinNonlin software (shown in Table 2). The mean plasma concentration-time curves of free quercetin in rats are shown in Figure 7. The AUC determines the bioavailability of the drug for the given the same dose in the formulation. The absolute bioavailability (F_abs_) was calculated as followed:

$$F_{\text{abs}}=({\text{AUC}}_{T}\cdot D_{i.v.})/({\text{AUC}}_{i.v.}\cdot D_{T})\times 100\%$$

**Table 2:** Que pharmacokinetic parameters of all Que formulations in rat plasma.

Groups	*T*_max_ (h）	*C*_max_ (ng/mL)	AUC_0→∞_ (ng/mL) *h	MRT_last_ (h)	F_abs_ (%)
Que Sol (*i.v.*)	0.29 ± 0.10*	1316.16 ± 658.77***	2489.35 ± 643.44***	5.28 ± 1.32	100
Que Suspension	0.53 ± 0.34	16.73 ± 6.33	89.93 ± 38.42	6.25 ± 3.11	3.61
TPGS-Que-NSps	0.61 ± 0.71	52.68 ± 16.87*	387.09 ± 60.28**	9.50 ± 4.42*	15.55
TPGS-SO-Que-NSps	1.33 ± 0.75	22.67 ± 7.71	172.74 ± 45.65*	7.87 ± 2.96	6.93
SPC-Que-NSps	1.06 ± 0.43	37.12 ± 2.01*	308.14 ± 58.31**	8.68 ± 1.56	12.38
SPC-Pip-Que-NSps	0.92 ± 0.15	24.61 ± 9.03	587.08 ± 109.05**	10.80 ± 3.22*	23.58

The results are presented as the mean ± SD, *n* = 6. *T*_max_: time to peak; *C*_max_: maximum concentration; AUC: area under the curve; MRT_last_: mean residence time; Fabs: absolute bioavailability. *p < .05, **p < .01, ***p < .001 versus Que suspension group.

where *T* represents the test group, *i.v.* represents the intravenous control group, and *D* stands for the dose.

As shown in Figure 7(a), quercetin plasma concentration of Que-NSps groups were all significantly higher than Que physical suspensions. The *C*_max_ value of free Que in TPGS-Que-NSps (52.68 ± 16.87 ng/mL) was the biggest among all groups. The *T*_max_ of Que for Que-NSps groups were all prolonged compared to Que suspensions. *T*_max_ for TPGS-Que-NSps and TPGS-SO-Que-NSps were respectively 1.2- and 2.5-folded than that of Que suspensions. *T*_max_ of SPC-Que-NSps and SPC-Pip-Que-NSps were, respectively, 2.0- and 1.7-folded than that of Que suspension.

The AUC_0→∞_ value of quercetin for TPGS-Que-NSps (387.09 ± 60.28 (ng/mL)*h) and TPGS-SO-Que-NSps (172.74 ± 45.65 (ng/mL)*h) were respectively 4.3 times (*p* < 0.01) and 1.9 times (*p* < 0.01) greater than Que suspension (89.93 ± 38.42 (ng/mL) *h). The AUC_0→∞_ value of quercetin for SPC-Que-NSps (308.14 ± 58.31 (ng/mL)*h) and SPC-Pip-Que-NSps (587.08 ± 109.05 (ng/mL)*h) were, respectively, 3.4 times (*p* < 0.01) and 6.5 times (*p* < 0.01) greater than Que suspension.

The mean plasma concentration–time curves of Que-glu and isorhamnetin in rats are shown in Figure 7(b,c). The *T*_max_, *C*_max_, and AUC_0→∞_ of Que-glu and isorhamnetin are listed in Table 3. The *T*_max_ of Que-glu and isorhamnetin was basically in accordance with that of quercetin. The concentration of isorhamnetin was always in a quite low level of all groups. However, the concentration of Que-glu was much higher than isorhamnetin or quercetin which preliminary confirmed the quick UGT metabolism of quercetin after absorbed into enterocyte and the bloodstream.

**Table 3.** Pharmacokinetic parameters of Que-glu and isorhamnetin all Que formulations in rat plasma.

	Groups	*T*_max_ (h)	*C*_max_ (ng/mL)	AUC_0→∞_ (ng/mL) *h
Que-glu	Que Sol (*i.v.*)	0.26 ± 0.13*	19,100.05 ± 4020.45***	17,298.20 ± 4777.45
	QUE Suspension	0.72 ± 0.51	248.25 ± 64.03	4375.87 ± 1731.04
	TPGS-Que-NSps	0.59 ± 0.36	989.5 ± 290.53*8	3528.40 ± 1532.63
	TPGS-SO-Que-NSps	0.18 ± 0.44*	580.71 ± 79.93*	1693.43 ± 523.41
	SPC-Que-NSps	0.12 ± 0.45*	1357.55 ± 483.14**	5690.16 ± 2054.23
	SPC-Pip-Que-NSps	0.27 ± 0.12*	164.53 ± 49.17	1735.62 ± 458.55
isorhamnetin	Que Sol (*i.v.*)	0.29 ± 0.23	1675.13 ± 113.45**8	2826.56 ± 465.69***
	QUE Suspension	0.28 ± 0.17	8.29 ± 4.64	229.84 ± 40.31
	TPGS-Que-NSps	0.13 ± 0.02	16.32 ± 6.49*	149.48 ± 63.72*
	TPGS-SO-Que-NSps	1.08 ± 1.48	7.14 ± 3.90	68.41 ± 33.26**
	SPC-Que-NSps	1.54 ± 0.73	15.06 ± 9.01*	271.63 ± 80.42
	SPC-Pip-Que-NSps	1.01 ± 3.12	7.25 ± 3.02	116.52 ± 31.48*

The results are presented as the mean ± SD, *n* = 6. *T*_max_: time to peak; *C*_max_: maximum concentration; AUC: area under the curve; MRT_last_: mean residence time; F_abs_: absolute bioavailability. *p < 0.05, **p < 0.01, ***p < 0.001 versus Que suspension group.

These results showed all the Que-NSps formulations can increased absorption of quercetin by oral administration. The improved bioavailability by nanosuspensions formulation might be ascribed to direct uptake of nanoparticles through the GI tract. Firstly, nanoparticles can increase the retention time in gastrointestinal mucosa, surfactants like TPGS and SPC can increased permeability and affinity between lipid particles and intestinal membrane so that more quercetin in nanosuspensions could be uptaken through GI tract wall and intestinal membrane. What’s more, TPGS can inhibit P-gp activity in the intestinal cell membrane and thus increase more cellular uptake of TPGS-Que-NSps, reduce drug efflux and prolong retention time of TPGS-Que-NSps (Mei et al., 2013). Secondly, the particle size played a leading role in absorption rate. The size of Que-NSps at around 200∼300 nm allowed more uptake in the lymphoid sections, therefore bypass the liver first-pass metabolism (Hussain et al., 2001; Tao and Desai, 2005; Yuan et al., 2013; Banerjee and Mitragotri, 2017). Thirdly, quercetin’s exposure to metabolic enzymes during absorption process was reduced by incorporation into nanoparticles. And the sustained drug release prolonged quercetin residence time in systematic circulation. There were two absorption peaks in the time-concentration curve of Que-NSps, the first absorption peak was the *C*_max_, which represented the released quercetin from nanosuspensions into the intestinal tract in the quick release period. The second peak of SPC-Que-NSps and SPC-Pip-Que-NSps occurred at 8 h, TPGS-Que-NSps and TPGS -SO-Que-NSps at 12 h. The second peak was smaller which may be caused by the slow-release period or reabsorption of quercetin hydrolyzed from metabolites like Que-glu.

As for Que-NSps with metabolic inhibitors SO or Pip, it seemed only Pip exerted metabolic inhibiting effect. The AUC_0→∞_ value of quercetin for SPC-Pip-Que-NSps was 1.9 times greater than SPC-Que-NSps. And the AUC_0→∞_ value of Que-glu of SPC-Pip-Que-NSps was significantly lower than SPC-Que-NSps (8.2 times lower). The decreased first pass glucuronidation was the main cause of enhanced oral absorption of quercetin. These results both proved Pip effectively inhibited quercetin from transforming to Que-glu formation. But the AUC_0→∞_ value of quercetin for TPGS-SO-Que-NSps was lower than TPGS-Que-NSps. The reasons were analyzed as followed: firstly, TPGS-SO-Que-NSps were less stable in gastrointestinal fluid (Figure 4(b)). Secondly, the amount of SO used in nanosuspensions may be not enough to exert metabolic inhibition effect. Thirdly, the release of quercetin was retarded due to aggregation when TPGS-SO-Que-NSps in gastrointestinal fluids so that the ideal metabolic inhibition effect of SO was not exhibited.

## Conclusion

In this research, we have successfully prepared different quercetin nanosuspensions stabilized by TPGS or SPC. Pip or SO was added to Que-NSps as metabolic inhibitors. All Que-NSps exhibited nanoscale spherical structure, good stability in gastrointestinal fluid *in vitro*. Pharmacokinetic studies revealed prolonged MRT, and improved absolute bioavailability of Que-NSps to Que suspension in rats after oral administration was observed. Pip demonstrated excellent metabolic inhibition effect on quercetin. These results collectively supported that metabolic inhibitors based on nanosuspensions is an effective solution to enhance oral absorption of quercetin.
